# Characterization of the *Liriodendron chinense* Pentatricopeptide Repeat (PPR) Gene Family and Its Role in Osmotic Stress Response

**DOI:** 10.3390/genes14061125

**Published:** 2023-05-23

**Authors:** Xiaoxiao Ma, Dandan Wang, Guoxia Xue, Xueyan Zheng, Ye Lu, Jisen Shi, Zhaodong Hao, Jinhui Chen

**Affiliations:** 1 State Key Laboratory of Tree Genetics and Breeding, Co-Innovation Center for Sustainable Forestry in Southern China, Nanjing Forestry University, Nanjing 210037, China; 2 Key Laboratory of Forest Genetics and Biotechnology, Ministry of Education, Nanjing Forestry University, Nanjing 210037, China; 3 National Germplasm Bank of Chinese Fir at Fujian Yangkou Forest Farm, Shunchang 353211, China

**Keywords:** drought stress, expression pattern, *Liriodendron chinense*, *PPR*

## Abstract

The *Pentatricopeptide repeat* (*PPR*) superfamily is a large gene family in plants that regulates organelle RNA metabolism, which is important for plant growth and development. However, a genome-wide analysis of the *PPR* gene family and its response to abiotic stress has not been reported for the relict woody plant *Liriodendron chinense*. In this paper, we identified 650 *PPR* genes from the *L. chinense* genome. A phylogenetic analysis showed that the *LcPPR* genes could roughly be divided into the P and PLS subfamilies. We found that 598 *LcPPR* genes were widely distributed across 19 chromosomes. An intraspecies synteny analysis indicated that duplicated genes from segmental duplication contributed to the expansion of the *LcPPR* gene family in the *L. chinense* genome. In addition, we verified the relative expression of *Lchi03277*, *Lchi06624*, *Lchi18566*, and *Lchi23489* in the roots, stems, and leaves and found that all four genes had the highest expression in the leaves. By simulating a drought treatment and quantitative reverse transcription PCR (qRT-PCR) analysis, we confirmed the drought-responsive transcriptional changes in four *LcPPR* genes, two of which responded to drought stress independent of endogenous ABA biosynthesis. Thus, our study provides a comprehensive analysis of the *L. chinense PPR* gene family. It contributes to research into their roles in this valuable tree species’ growth, development, and stress resistance.

## 1. Introduction

The *pentatricopeptide repeat* (*PPR*) superfamily is one of the largest gene families in plants. Typically, PPR proteins comprise more than two tandem repeats of conservative motifs containing 30–40 amino acids [1,2]. PPR proteins can be classified into P and PLS subfamilies, based on the members’ motif structure. The PLS subfamily possesses P1, P2, L1, L2, S1, S2, SS, E1, or E2 motifs and occasionally DYW, a non-PPR motif, whereas members of subfamily P only possess tandem P motifs [2]. The PLS subfamily protein is unique to terrestrial plants and was first discovered in *Physcomitrium patens* [2]. According to available research, most PPR proteins have distinct binding sites, leading to minimal functional redundancy across multiple PPR members [3]. Compared with the hundreds of PPR members in the species, the functionally identified PPR proteins remain a minority. Most PPR proteins target mitochondria or chloroplasts; some PPR proteins have been proven to be dual-targeted [4]. PPR proteins primarily control organelle RNA metabolism such as RNA editing [5,6], RNA splicing [7], and RNA translation [8].

PPR proteins play a crucial role in cytoplasmic male sterility [9] and embryo development [10]. Mitochondrial-targeted PPR proteins regulate proper embryonic development and help prevent cytoplasmic male sterility (CMS) [11,12,13]. Mitochondrial-localized PPR proteins’ loss of function usually results in abnormal embryonic development [14,15]. The deletion mutant of AtPPR protein OGR1 has demonstrated sluggish growth, late flowering, plant dwarfing, and sterility due to defective RNA editing [16]. Restorer of fertility (Rf) PPR proteins repress the expression of mitochondrial transcripts that cause infertility, and their deletion mutants cannot produce functional pollen. At present, the *Rf* gene has been successfully extracted from *Petunia parodii* [17], *Capsicum annuum* [18], *Arabidopsis thaliana* [19], *Oryza sativa* [20], and other plants. A defect in chloroplast-targeted PPR proteins often results in embryo abortion [21,22]. Although it can sometimes grow into a plant, it will eventually die due to the chloroplast function’s failure to obtain nutrients [23].

Additionally, PPR proteins are crucial for plants to respond to biotic or abiotic stressors [24]. For example, the *SLO2* gene in *Arabidopsis* was found to be involved in plant energy metabolism and stress response [25,26], whilst *slo2* mutants have exhibited a phenotype of stunted growth and restricted root development [25,26]. Furthermore, *slo2* has also induced ABA hypersensitivity and ethylene insensitivity in plants [25,26]. The *Arabidopsis SOAR1* overexpression line has greatly improved resistance to cold, salt, and drought stresses, and its seeds can even sprout and thrive in seawater [27]. *Arabidopsis* mutants of *ppr40* showed plant dwarfing and increased sensitivity to salt, abscisic acid, and oxidative stresses [28]. *PPS1* is involved in balancing reactive oxygen species (ROS), which has an important role in plant responses to abiotic stresses [29], whereas the *pps1* mutant exhibited enhanced sensitivity to salt stress and ABA [29]. *AtPPR96* is involved in the response of plants to salt stress [30].

All terrestrial plants contain many PPR proteins [9]; for example, *Arabidopsis thaliana* has 450 PPR proteins and *Populus trichocarpa* has 626 PPR proteins [1,31]. Although the evolutionary status greatly varies, *Physcomitrium patens* and *Arabidopsis thaliana* have similar exon sequences, but the former contains more introns in its gene structure. As these homologous proteins may have similar functions, researchers have hypothesized that intron-rich PPR genes might be ancestral types and that many intron-free genes have evolved through reverse transcriptional transposition events [32,33,34]. Additionally, recent research has determined that the *PPR* gene family underwent expansion events, mostly in the form of genome segmental duplication, prior to the differentiation of the euphyllophytes and the lycophytes [35].

*Liriodendron* belongs to the Magnoliaceae family, which is an early-diverging angiosperm family with a special evolutionary status [36]. The study of the *LcPPR* gene family in *L. chinense* helps us to better understand the evolution and expansion of *PPR* genes in angiosperms. In addition, low natural fruiting rates threaten the natural population size of *L. chinense* [37]. However, with the establishment of a mature transgenic system and somatic embryogenesis system [38], research on this species has been greatly facilitated. By studying the *LcPPR* gene, we hope to find relevant genes that can improve *L. chinense* resistance to the environment and further apply them in production practices to promote the conservation and research of this species. In this study, we identified the *LcPPR* gene family in the *L. chinense* genome and performed gene structure analyses, phylogenetic analyses, and gene duplication analyses. Furthermore, we explored the expression profiles of four *LcPPR* genes under drought stresses based on transcriptome data [39] and validated their relative expression using quantitative reverse transcription PCR (qRT-PCR). These findings serve as a reference point for clarifying the role of *LcPPR* genes and provide a foundation for future genetic modifications.

## 2. Materials and Methods

### 2.1. Plant Materials and Stress Treatment

In this study, three-month-old seedlings of *Liriodendron hybrid* were used for tissue-specific expression patterns; roots, stems, and leaves were sampled in three biological replicates, respectively. For the stress treatment, three-month-old plants of a similar growth status were separately treated with 20% PEG, 50 mg/L ABA, 20% PEG, and 50 µM fluridone. The leaves were collected at 0, 12, and 24 h after treatment, with three biological replicates each. For the qRT-PCR tests, all samples were instantly frozen in liquid nitrogen and stored at −80 °C. The growth conditions were 22 °C, with a 16 h of light and 8 h of dark photoperiod. The relative humidity was 75% [39].

### 2.2. Identification of PPR Genes in L. chinense

We used the PPR (PF01535) seed file downloaded from the Pfam (https://pfam.xfam.org/, accessed on 5 May 2022) database and HMMER3.0 software to search for the genome of *L. chinense*. We also performed a BlastP search for the *L. chinense* protein. Combining the aforementioned search results, we removed any unnecessary sequences. To confirm the remaining sequences, we employed SMART (http://smart.embl-heidelberg.de/, accessed on 6 May 2022) and CDD search tools (https://www.ncbi.nlm.nih.gov/Structure/bwrpsb/bwrpsb.cgi, accessed on 6 May 2022). After analyzing the sequence using the online HMMER search program (https://www.ebi.ac.uk/Tools/hmmer/, accessed on 8 May 2022), we removed proteins with less than two PPR motifs.

### 2.3. Sequence Analysis of LcPPR and Phylogenetic Tree Construction

We used Weblogo (http://weblogo.berkeley.edu/logo.cgi, accessed on 8 May 2022) to create a motif identification to evaluate the conservatism of the *LcPPR* motif. We used the TBtools (v1.108) program to display data after retrieving each gene’s chromosomal and exon/intron locations from the *Liriodendron* database [40]. We also used the TBtools (v1.108) program to calculate Ka/Ks values. To investigate the collinearity of the *LcPPR* genes, we employed a multi-collinearity scanning tool package. Additionally, we used ExPASy ProtParam (https://web.expasy.org/protparam/, accessed on 9 May 2022) to determine the theoretical isoelectric point, molecular weight, and protein hydrophilicity of *LcPPR* genes. We conducted a multi-alignment of 650 LcPPR proteins using MAFFT software (v7.037). The tree was constructed using the maximum likelihood (ML) method with FastTree software (v2.1.10) and a bootstrap analysis of 1000 replicates. The diagram was drawn and modified via iTOL (https://itol.embl.de/itol.cgi, accessed on 4 April 2023).

### 2.4. Cis-Acting Element Prediction

We extracted a 2500 bp sequence upstream of the initiation codon of the target gene from the *Liriodendron* genome and used PlantCARE databases (http://bioinformatics.psb.ugent.be/webtools/plantcare/html/, accessed on 20 May 2022) to analyze the sequence for potential cis-acting elements. Finally, the results were visualized using TBtools software (v1.108).

### 2.5. Expression Analysis of LcPPR Genes in L. chinense

We performed tissue-specific and drought stress expression analyses using published transcriptome data from the NCBI Sequence Read Archive (SRA) database with the following accession numbers: PRJNA559687 (bract, petal, sepal, stamen, pistil, leaf, and shoot apex) [36] and PRJNA679101 (drought) [39]. The raw data were analyzed using the same process as Wu et al. [39]. A differential expression analysis of transcriptomic data was performed using the edgeR package with a screening condition of log_2_ (fold change) > 1 and FDR < 0.05. We then used TBtools to plot a heatmap to visualize the differential gene expression levels. We conducted a qRT-PCR analysis to validate the transcriptome data and determine the expression pattern for selected genes after ABA, PEG, PEG, and fluridone treatments. The total RNA was isolated using an RNA extraction kit (Promega, Shanghai, China, LS1040), and cDNA was synthesized through a reverse transcription kit (Vazyme, Nanjing, China, R312-02). We used the Livak calculation method to calculate the genes’ expression levels based on three technical and biological duplicates [41]. For qPCR data, 2^−ΔΔCt^ was used for the statistical analysis. We normalized them with the reference genes *α-Tubulin* and *GAPDH* as well as 18S rRNA [42]. Table S1 lists the primers for qRT-PCR.

We used IBM SPSS Statistics 26 for the statistical analysis. Data were analyzed using a one-way ANOVA with Tambane’s T2 analysis or Tukey’s correction for multiple comparisons. Values were stated as the mean ± standard deviation. In all comparisons, *p* < 0.05 was considered to be statistically significant. We used GraphPad Prism 8 to visualize our results.

## 3. Results

### 3.1. Genome-Wide Identification of the LcPPR Gene Family in L. chinense

**Figure 1:**
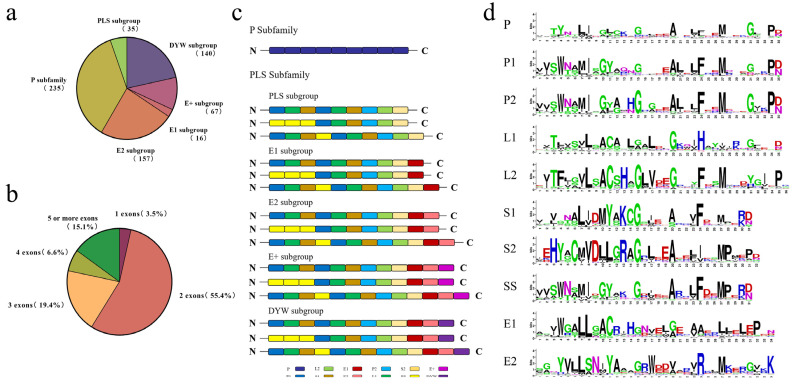
The genome-wide identification and structural analysis of the *LcPPR* gene family in *L. chinense*: (**a**) classification of LcPPR proteins and the number of members in each category; (**b**) exon number distribution of *LcPPR* genes; (**c**) typical pattern organization of different categories of LcPPR proteins; (**d**) amino acid arrangement of each conserved structural domain of LcPPR proteins. Letters are abbreviations of amino acid names, and their size indicates the conservativeness of the amino acid. The larger the letter, the higher the conservativeness.

Firstly, we identified 705 *PPR* candidate genes for *L. chinense* using a similarity-based search. Then, by examining the conserved domain, we confirmed 650 *LcPPR* genes (Table S2 and Figure 1a). The molecular weight (MW) of the LcPPR proteins varied from 16,623.22 to 219,313.38 Da. The theoretical isoelectric point (pI) ranged from 4.51 to 9.67, and the sequence length spanned from 149 to 1911 amino acids. In addition, 501 LcPPR proteins had GRAVY values less than zero, suggesting that they were hydrophilic proteins. According to the gene structure analysis, 3.5% (23/650) of them had just one exon, whereas 55.4% (360/650), 19.4% (126/650), 6.6% (43/650), and 15.1% (98/650) had two, three, four, and more than five exons, respectively (Figure 1b and Figure S1). Based on the analysis of the repeated motif structure, the LcPPR proteins could be divided into P and PLS subfamilies. The former contained only the P motif (235 members), whereas the latter contained several P1, P2, L1, L2, S1, S2, SS, E1, E2, and DYW motifs arranged in a specific order. The PLS subfamily could be categorized into E1 (16 members), E2 (157 members), E+ (67 members), and DYW (140 members) subgroups, depending on the carbon terminal structural domain (Figure 1c). The number of repeated motifs per LcPPR protein ranged from 2 to 39. Figure 1d shows the amino acid arrangement of each conserved domain of LcPPR proteins.

### 3.2. Phylogenetic Analysis of LcPPR Proteins

**Figure 2:**
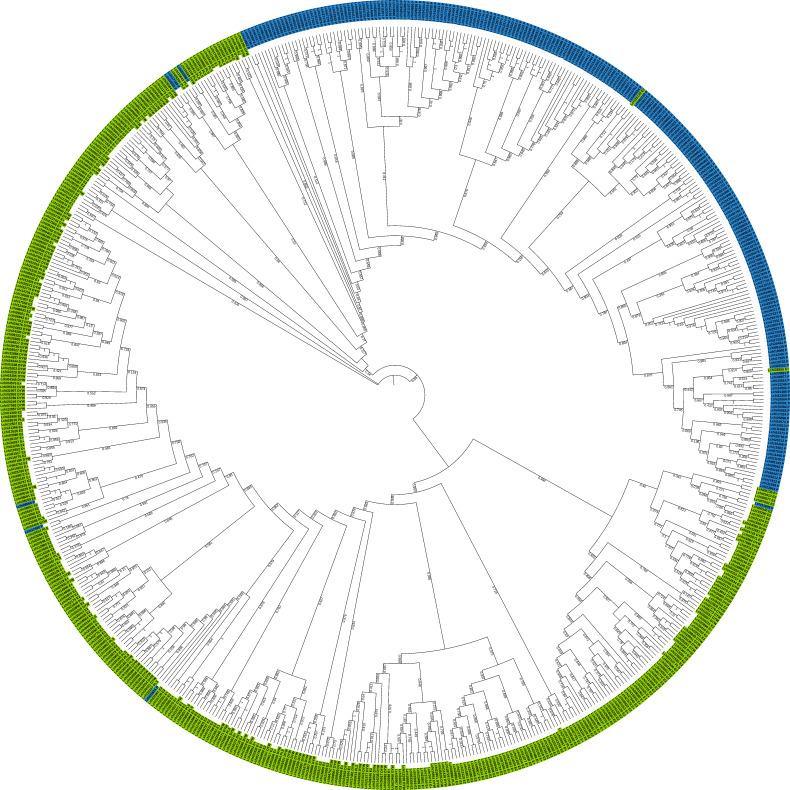
Phylogenetic analysis of the *PPR* genes in *L. chinense*. MAFFT aligned the full sequences of 650 LcPPR proteins, and the phylogenetic tree was constructed using FastTree with the maximum likelihood method and 1000 bootstrap replicates. Blue represents members of the P subfamily, and green represents members of the PLS subfamily.

To comprehend the evolutionary relationships between these LcPPR proteins, we constructed a phylogenetic tree using the 650 LcPPR proteins’ full-length amino acid sequences. Our results showed that group members of the P and PLS subfamilies were clearly separated (Figure 2). Notably, seven members (*Lchi01299*, *Lchi17169*, *Lchi24030*, *Lchi08060*, *Lchi17690*, *Lchi23616*, and *Lchi11005*) of the P subfamily were clustered with members of the PLS subfamily, whereas *Lchi26149* and *Lchi02855* in the PLS subfamily were clustered with branches of the P subfamily. The clustering of members in each subgroup of the PLS subfamily was not satisfactory, and only PLS subgroup members clustered relatively well.

### 3.3. Chromosomal Distribution and Duplication of LcPPR Genes

**Figure 3:**
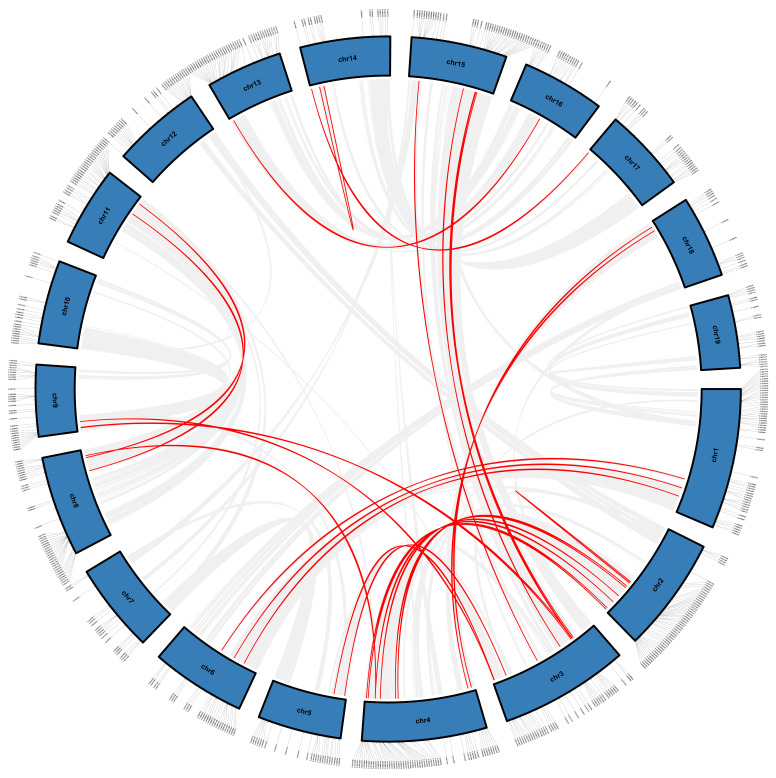
The chromosomal distribution and gene duplication of *LcPPR* in the *L. chinense* genome. The solid line at the edge of the blue box indicates the gene’s location, and the red line inside the circle indicates the gene pair where duplication occurred.

We found 598 irregularly and extensively dispersed *LcPPR* genes throughout 19 pseudo-chromosomes of *L. chinense*, whereas the remaining 52 members could not be assigned to the pseudo-chromosomes (Table S2). Most *LcPPR* genes were located on chromosome 4 (55 members), whereas the fewest were found on chromosome 18 (14 members). An intraspecies collinearity analysis showed that 30 pairs of *LcPPR* genes within collinear blocks existed in the *L. chinense* genome (Figure 3), indicating that duplicated genes from segmental duplication functioned in expanding the *LcPPR* gene family. The highest number of gene duplication events occurred on chromosomes 2, 3, and 4. To analyze the selective pressure on the *LcPPR* gene family, we calculated Ka and Ks distance values for the 30 pairs of *LcPPR* genes described above. The results revealed 27 collinear gene pairs with Ka/Ks values less than 1 (Table S3).

### 3.4. Expression Patterns of LcPPR Genes in Different Tissues

**Figure 4:**
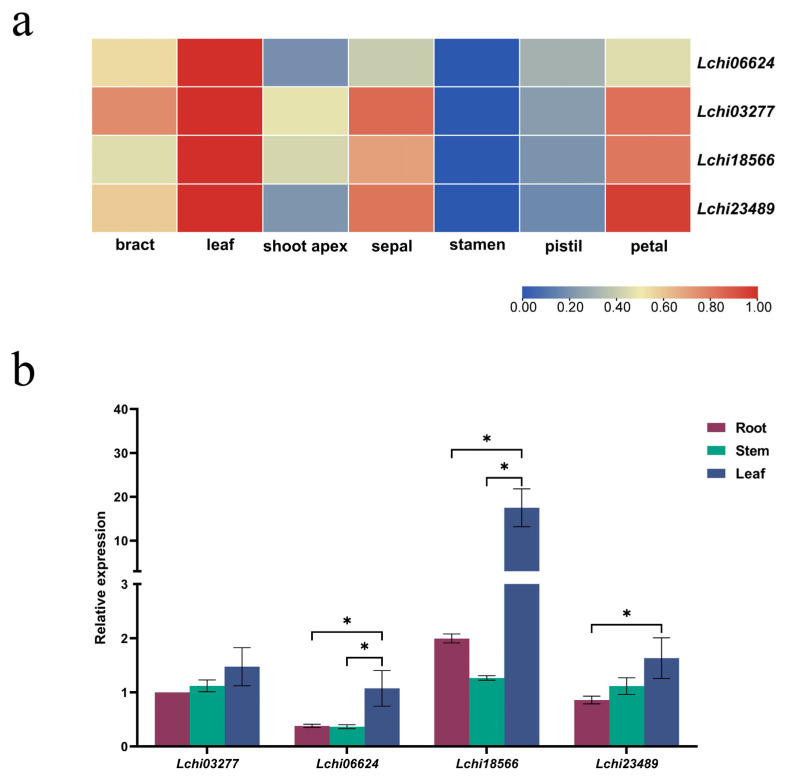
Relative expression of four *LcPPR* genes in different tissues of *L. chinense*. (**a**) Expression profiles of four *LcPPR* genes based on transcriptome data. The FPKM values were used to indicate gene expression levels [43]; (**b**) qRT-PCR analysis of *LcPPR* genes in the root, stem, and leaf of *L. chinense*. *Lchi03277* expression in the root was used as a control to determine the relative expression levels. The y-axis shows the expression level (2^−ΔΔCt^). Asterisks indicate statistically significant differences. * *p* < 0.05.

Expression patterns of genes in different tissues may correlate with gene function. We first mapped the expression patterns of all *LcPPR* genes in the bract, leaf, shoot apex, sepal, stamen, pistil, and petal based on the transcriptome data and found that the *LcPPR* genes had a variety of expression patterns (Figure S2). Using a differential expression analysis, we screened 20 *LcPPR* genes that had significantly different expression levels in leaves (Figure S3). Four of them were selected using qRT-PCR to verify their relative expressions in the root, stem, and leaf of *L. chinense* (Figure 4a). Figure S4 shows the results of the specific qRT-PCR primer detection. The melting curve for the validated genes is shown in Figure S5. All these genes were more highly expressed in the leaves, whereas similar levels were expressed in the roots and stems (Figure 4b). *Lchi03277* and *Lchi23489* had similar expression levels and expression patterns. *Lchi06624* and *Lchi18566* were significantly more expressed in the leaves than in the roots and stems. *Lchi185666* was expressed at the highest level in the leaves, whereas *Lchi06624* was expressed at low levels in various tissues.

### 3.5. Analysis of the Cis-Acting Elements in LcPPR Gene Promoters

**Figure 5:**
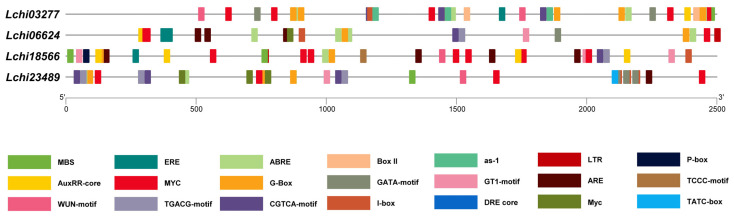
Cis-acting element analysis of four *LcPPR* genes in *L. chinense*.

The cis-acting elements of a promoter are tightly correlated with stress responses and gene transcription. Therefore, we further analyzed the promoter sequences about 2500 bp upstream of the initiator codon of four *LcPPR* genes. We found many elements, but focused on the cis-acting elements associated with plant hormones and environmental factors. The promoters of these four *LcPPR* genes all contained elements mentioned above such as the abscisic acid response element ABRE, the drought stress-related element MYC, MBS, and as-1. They indicated that abscisic acid could regulate these genes and be affected by drought stress (Figure 5 and Table 1).

### 3.6. Expression Analysis of LcPPR Genes under Drought Stresses

**Figure 6:**
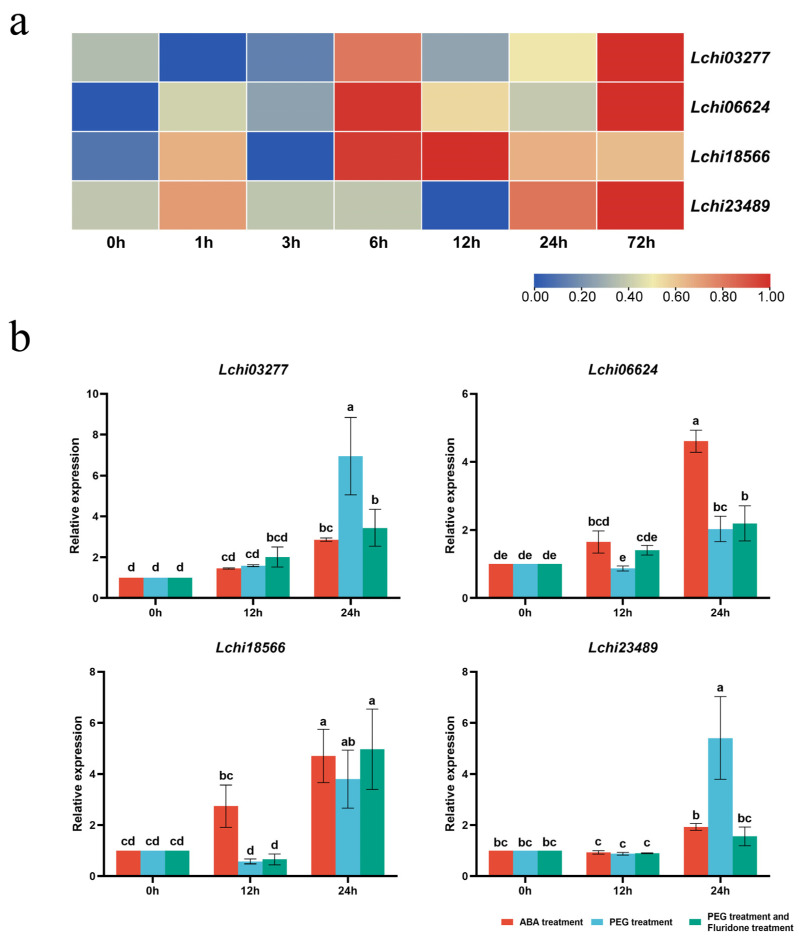
Relative expression of four *LcPPR* genes under drought stress. (**a**) Expression profiles of *LcPPR* genes under drought stress based on transcriptome data. The FPKM values were used to indicate gene expression levels; (**b**) qRT-PCR analysis of *LcPPR* genes under ABA, PEG, and fluridone treatment. The expression of each gene at 0 h was used as a control to determine the relative expression level. A one-way ANOVA test was used to analyze the data. Letters a–e were used to label statistical significance. There was no statistical significance between groups containing the same letter (*p* < 0.05).

Based on the transcriptome data [39], we explored the expression profiles of four *LcPPR* genes under drought stress. All genes were upregulated to varying degrees after 72 h in response to drought stress, suggesting their involvement in drought resistance in *L. chinense* (Figure 6a).

To confirm our speculation, we treated the three-month-old plants with ABA, PEG, PEG, and fluridone. The qRT-PCR results showed that all four genes were transcriptionally upregulated in response to either the ABA or PEG treatment (Figure 6b). The transcriptional responses of *Lchi03277* and *Lchi23489* were partially dependent on endogenous ABA biosynthesis because the fluridone treatment significantly decreased the upregulation of these genes in response to the PEG treatment at 24 h. However, an exogenous ABA treatment could not completely simulate the effects of the PEG treatment for *Lchi03277* and *Lchi23489*. In addition, *Lchi06624* appeared to be more sensitive to the ABA than the PEG treatment, and its response to the PEG treatment was independent of endogenous ABA biosynthesis. Meanwhile, *Lchi18566* also responded to the PEG treatment independently of ABA.

## 4. Discussion

With the completed genome sequencing of more species, the identification of *PPR* gene family members has significantly progressed. At present, in addition to model plants such as *Arabidopsis thaliana* [1], *Oryza sativa* [24], and *Populus trichocarpa* [31], *PPR* gene families have been identified in *Physcomitrium patens* [44], *Gossypium* [45], *Citrullus lanatus* [46], *Camellia sinensis* [47], and other plants. However, little is known about woody plants, especially magnoliids. We identified 650 *LcPPR* genes in the genome assembly of *Liriodendron chinense*, a relict woody magnoliid species. The number of *PPR* genes in *L. chinense* is higher than in herbaceous plants such as *A. thaliana* (450 members) [1] and *C. lanatus* (422 members) [46], but comparable with woody plants such as *P. trichocarpa* (626 members) [31] and *C. sinensis* (578 members) [47]. The presence of more *PPR* genes in woody plants than herbaceous plants indicates that they are more diverse, and may assume more functions in woody plants.

Based on the conserved structural domain alignment analysis, the *LcPPR* genes could be categorized into two subfamilies, P and PLS, which was also confirmed by the phylogenetic analysis. However, a few members in each subfamily were incorrectly grouped, as indicated by *P. trichocarpa* and *C. sinensis*. Consequently, several identical and conserved sites between the P motif and its variants could create ambiguities in the phylogenetic grouping. Based on the analysis of conserved structural domains, the amino acid arrangement of LcPPR proteins’ structural domains was highly consistent with other species [2], except that the antepenultimate site in E2 was lysine (V) in *L. chinense* instead of isoleucine (I) (Figure 1d). This finding indicated that PPR proteins are evolutionarily highly conserved. They may have important functions that could be severely affected by a mutation event.

Previous studies have shown that PPR proteins in angiosperms typically contain about 400–600 amino acid residues, and more than half of the individuals in most species do not have introns [24,31,48]. However, most *LcPPR* genes contain introns, and only 3.5% of the *LcPPR* genes are intron-free, with the highest number of cases containing one intron at 55.4%. Meanwhile, the coding sequence lengths showed no significant difference between *L. chinense* and *A. thaliana*, *P. trichocarpa*, and *C. sinensis* (Figure S6), indicating that intron variations largely caused the gene differentiation in *PPR* between different species. According to earlier research, most *PPR* genes eventually lose their introns due to amplification by retro-transposition events [32]. The gene structure analysis demonstrated that most *LcPPR* genes were ancestral types, consistent with the phylogenetic position of *L. chinense* in angiosperms [36].

Gene duplication is one of the main methods by which genes obtain new functions or expression patterns, which can promote the evolution of species [49]. In our study, the *LcPPR* genes were distributed on all chromosomes, and most were located in intraspecies collinear blocks. In addition, there were 30 duplicated gene pairs within the *LcPPR* gene family, suggesting that gene duplication caused by segmental genome duplication may be responsible for expanding the number of members in the *LcPPR* gene family. The results of a selective evolutionary analysis indicated that the *LcPPR* genes were under strong selective pressure for purification.

Tissue-specific studies of genes can provide a foundation for the functional exploration of genes. We selected four genes that were significantly expressed in the leaves by performing a differential expression analysis with transcriptome data. The qRT-PCR results showed that these genes were mainly expressed in the leaf, whereas the expression levels in the root and stem were similar. This finding may be because their function is mainly in the leaves and, to a lesser extent, in the roots and stems. To further determine these genes’ possible functions, we analyzed their promoter regions and identified several cis-acting elements that responded to environmental factors and phytohormones. We noticed that all four genes contained cis-acting elements in response to drought stress and ABA. Therefore, we focused on these genes’ expression patterns under drought stress in abiotic stress transcriptome data [39]. We found that all four genes were upregulated after 72 h of drought stress. Our results showed that these genes were indeed altered by drought stress, allowing us to further explore these genes’ role in drought stress.

We used a PEG treatment to simulate drought stress and found that all four genes appeared to be upregulated. Among them, *Lchi03277* was the most upregulated and affected by drought stress, whereas *Lchi06624* was the least affected by drought stress, with only a doubling of the upregulation expression. It is known that drought stress stimulates ABA production in plants [50]. Accumulated ABA can activate downstream genes to improve drought resistance in plants by closing the stomata and other pathways [50,51].

The exogenous application of ABA can promote the overexpression of genes and, consequently, enhance plant tolerance to stress [52]. In our study, the exogenous application of ABA promoted the expression of four *LcPPR* genes. *Lchi06624* and *Lchi18566* were more sensitive to the ABA treatment, with more than a four-fold expression. *Lchi23489* was the least responsive to exogenous ABA applications, with less than two-fold upregulation. We learned that both ABA and PEG applications could affect the expression of *LcPPR* genes. Therefore, we simultaneously applied PEG and fluridone to understand whether the *LcPPR* response to drought stress was related to endogenous ABA. *Lchi03277* and *Lchi23489* expressions decreased after the fluridone application compared with PEG alone, indicating that these genes’ upregulated expression levels were affected by endogenous ABA. Conversely, the expressions of *Lchi06624* and *Lchi18566* did not significantly change after applying fluridone, suggesting that endogenous ABA did not influence these two genes’ response to drought stress. *Lchi06624* and *Lchi18566* could respond to drought stress through an ABA non-dependent pathway. We may subsequently investigate the function of these two genes in other pathways such as the ROS pathway.

There were shortcomings in our experiments. One was that we could not treat the limited number of experimental materials to elucidate the effects on *LcPPR* genes under simultaneous drought stress and ABA induction. The second was that the number of genes validated by the experiment was not large enough, and many other interesting genes are waiting for further study.

## 5. Conclusions

In this study, we identified 650 *LcPPR* genes in the *L. chinense* genome, which could be divided into two subfamilies, P and PLS. Gene duplication, possibly from a WGD event, partially contributed to the expansion of the *LcPPR* gene family in *L. chinense*. We used transcriptome data to analyze the expression patterns of *LcPPR* genes in different tissues and under drought stresses. We then selected four genes for an experimental validation. Two of these genes could respond to drought stress through the ABA-independent pathway, whereas the other two responded through the ABA-dependent pathway. In summary, our results can guide further studies on *LcPPR* gene functions, especially in response to drought stress in *L. chinense*, thus promoting resistance breeding of this valuable woody tree.

## Figures and Tables

**Table 1:** Statistics on hormone- and environment-related cis-acting elements in four *LcPPR* genes.

			*Lchi03277*	*Lchi06624*	*Lchi18566*	*Lchi23489*
Hormone-Related	MeJA	CGTCA motif	3	1	1	3
		TGACG motif	3	1	1	3
	Auxin	AuxRR core	1	1	4	0
	Gibberellin	TATC box	0	0	0	1
		GARE motif	0	0	0	0
		P-box	0	0	1	0
	ABA	ABRE	7	5	1	4
	Ethylene	ERE	1	2	1	0
Environment-Related	Light	G-box	10	4	2	6
		Box II	2	0	0	0
		GATA motif	2	1	0	3
		I-box	1	1	1	6
		GT1 motif	0	1	3	1
		TCCC motif	0	0	1	0
		ATCT motif	0	0	0	0
	Wound	WUN motif	2	0	1	1
	Anaerobic induction	ARE	0	4	4	1
	Drought	MBS	1	0	2	1
		MYC	6	4	8	7
		DRE core	0	1	0	1
		as-1	3	1	1	3
	Low temperature	LTR	0	1	1	0

## Data Availability

The data and results are available to every reader upon reasonable request.

## Supplementary Materials

The following supporting information can be downloaded at: https://www.mdpi.com/article/10.3390/genes14061125/s1. Figure S1: Gene structure of *LcPPR* genes; Figure S2: Expression patterns of *LcPPR* genes in different tissues; Figure S3: Expression profiles of 20 *LcPPR* genes with significant expression in leaves; Figure S4: Specific detection of qRT-PCR primers; Figure S5: The melting curve for the validated genes; Figure S6: Average length of CDS Sequences in *Liriodendron chinense*, *Arabidopsis thaliana*, *Populus trichocarpa*, and *Camellia sinensis*; Table S1: Primers used for qRT-PCR; Table S2: Detailed information on *LcPPR* genes; Table S3: Ka and KS values.
