# Supplementary material for: Characterization of the Liriodendron chinense Pentatricopeptide Repeat (PPR) Gene Family and Its Role in Osmotic Stress Response
Source: Genes (Basel). 2023 May 23;14(6):1125. doi: 10.3390/genes14061125 (PMC10297974; doi:10.3390/genes14061125)
Supplement: Supplementary file 1 [file genes-14-01125-s001.zip › Table S1.pdf]

Table S1. Primers used for qRT-PCR

Gene name		Primer sequence
Lchi03277	F	TGTCGGGGCTTATAATGCTT
	R	GCACAAAATCCTTCTTGACCA
Lchi06624	F	AGCTCTTGCCTACTTCAACGA
	R	CTTTCATCCTTTGCACTAGCTT
Lchi18566	F	CACAATGAAAGATGGCCCAA
	R	GGATAACAACCCGCATCGAC
Lchi23489	F	AGCTCTTTGCATCTTCCGTCAG
	R	CCCATTCCTTCAAACACCTCGAA
α-Tubulin	F	CAGCTTGAGCGTGTCAATGT
	R	GATCAGTTCCGCTCCTTCAG
GAPDH	F	ACAACTAACTGCCTTGCTCCTT
	R	AGTCAGATCCACCACCGAAA
18S RNA	F	ATTTCTGCCCTATCAACTTTCG
	R	TTGTTATTTATTGTCACTACCTCCC
