# Supplementary figures and images for: Characterization of the Liriodendron chinense Pentatricopeptide Repeat (PPR) Gene Family and Its Role in Osmotic Stress Response
Source: Genes (Basel). 2023 May 23;14(6):1125. doi: 10.3390/genes14061125 (PMC10297974; doi:10.3390/genes14061125)

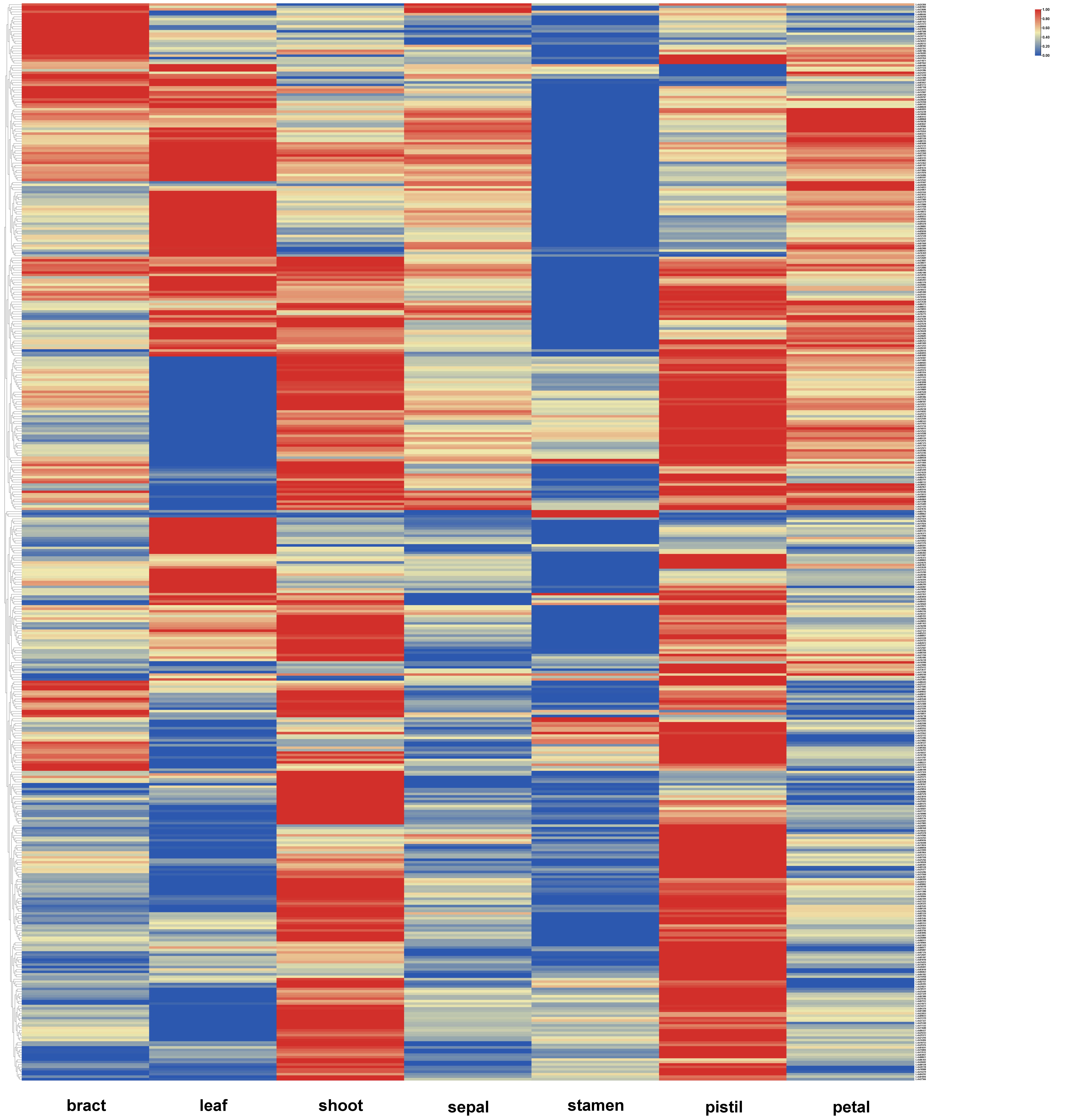

Supplement: Supplementary file 1 [file genes-14-01125-s001.zip › Figure S2.jpg]

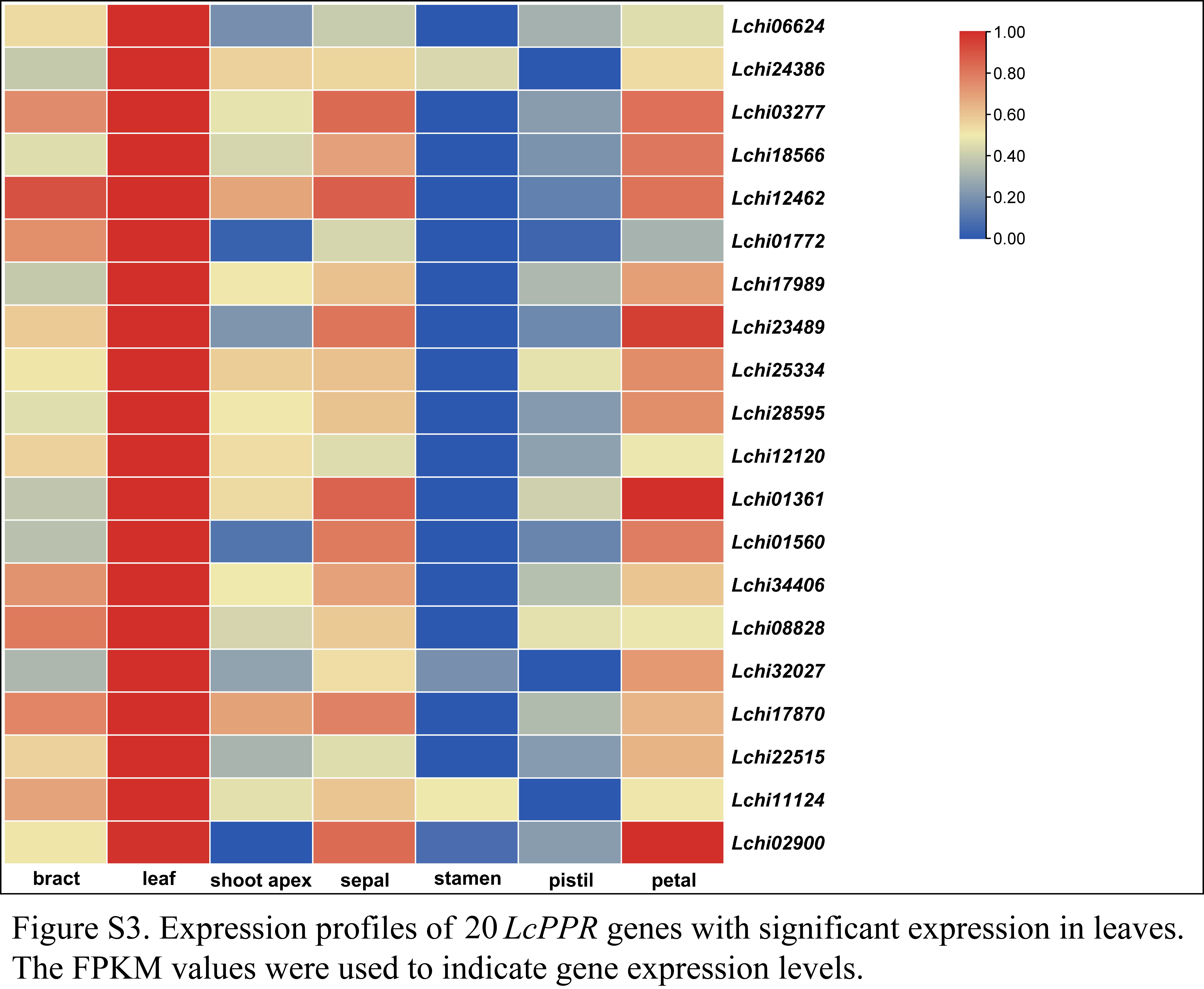

Supplement: Supplementary file 1 [file genes-14-01125-s001.zip › Figure S3.jpg]

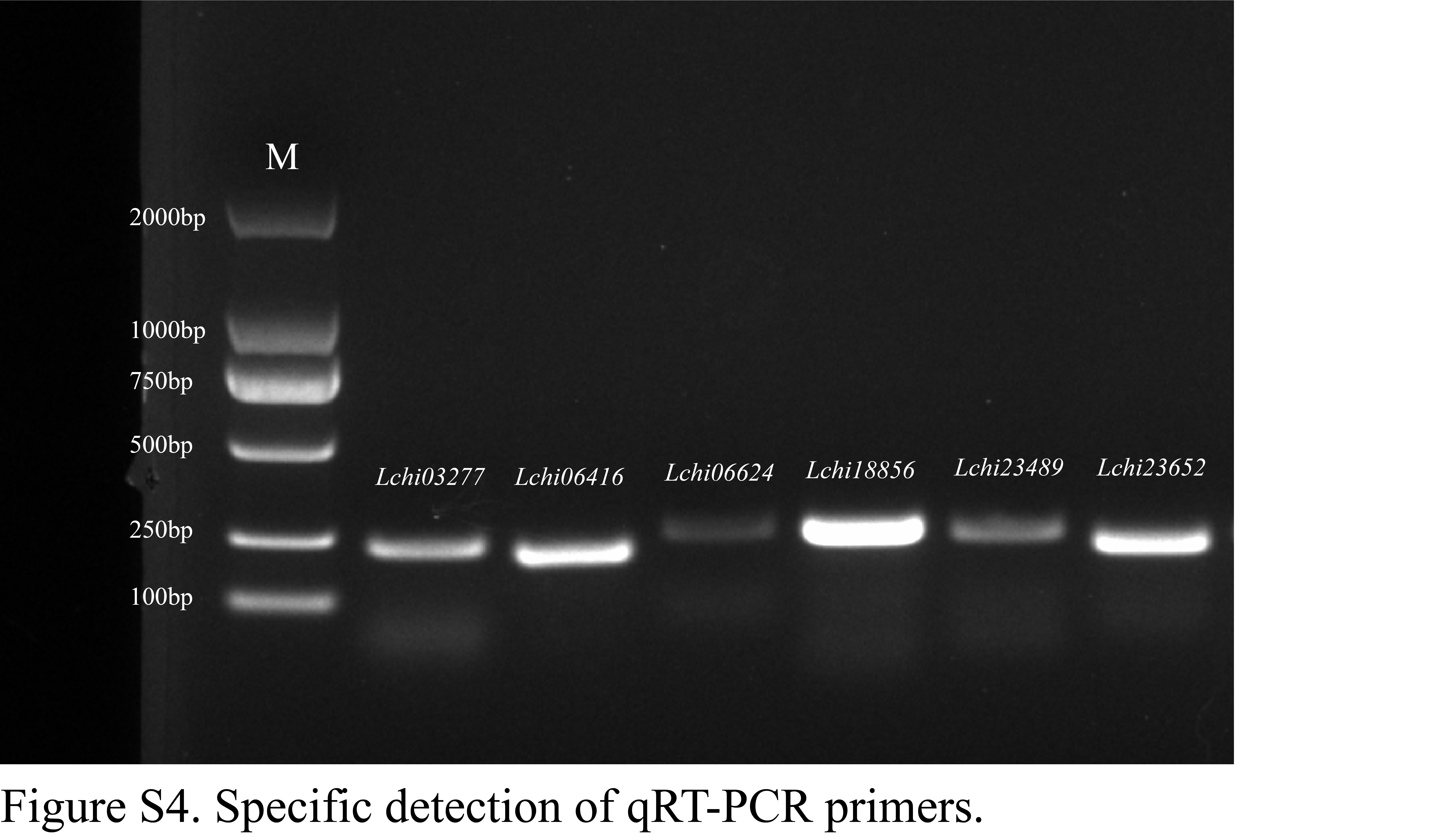

Supplement: Supplementary file 1 [file genes-14-01125-s001.zip › Figure S4.jpg]

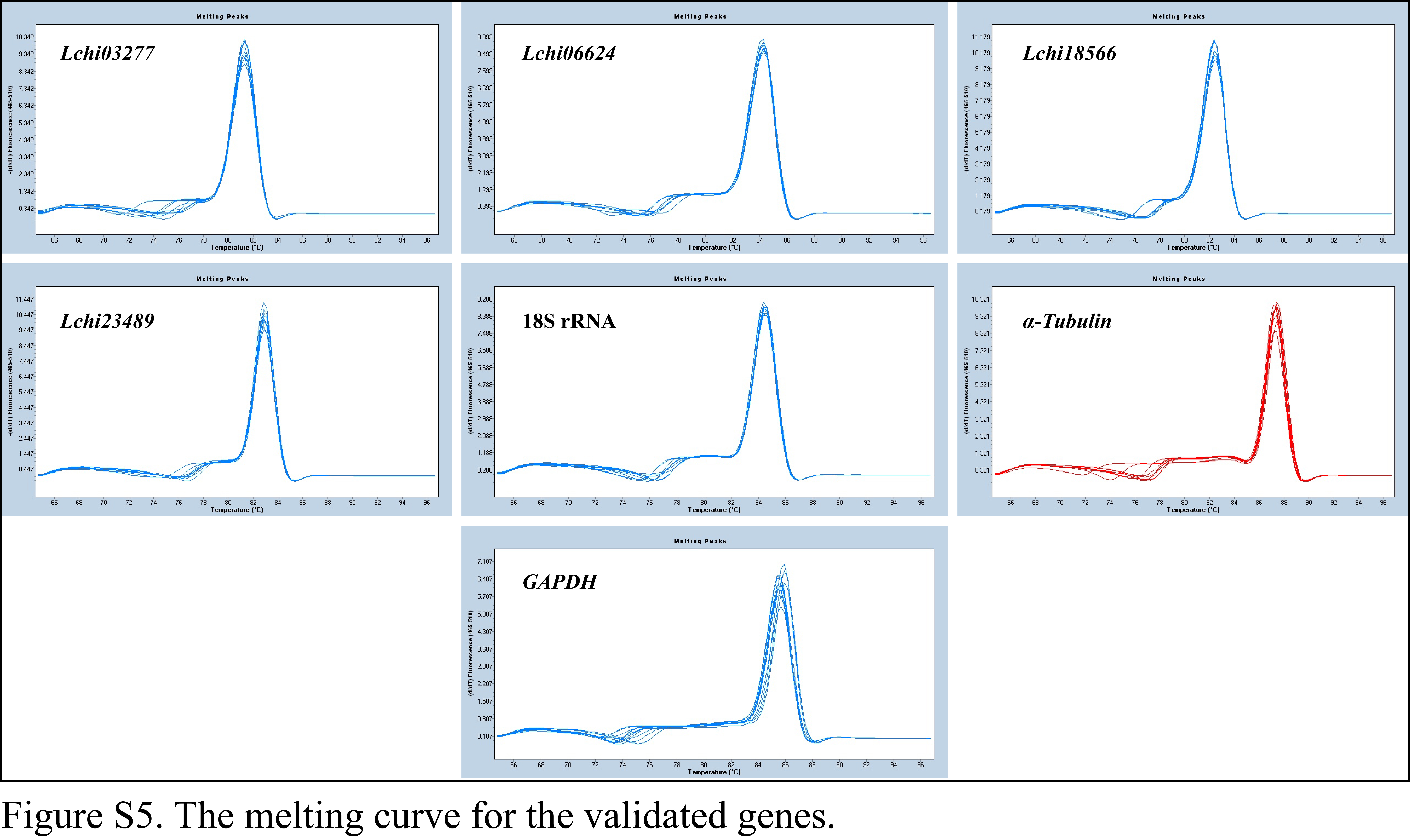

Supplement: Supplementary file 1 [file genes-14-01125-s001.zip › Figure S5.jpg]

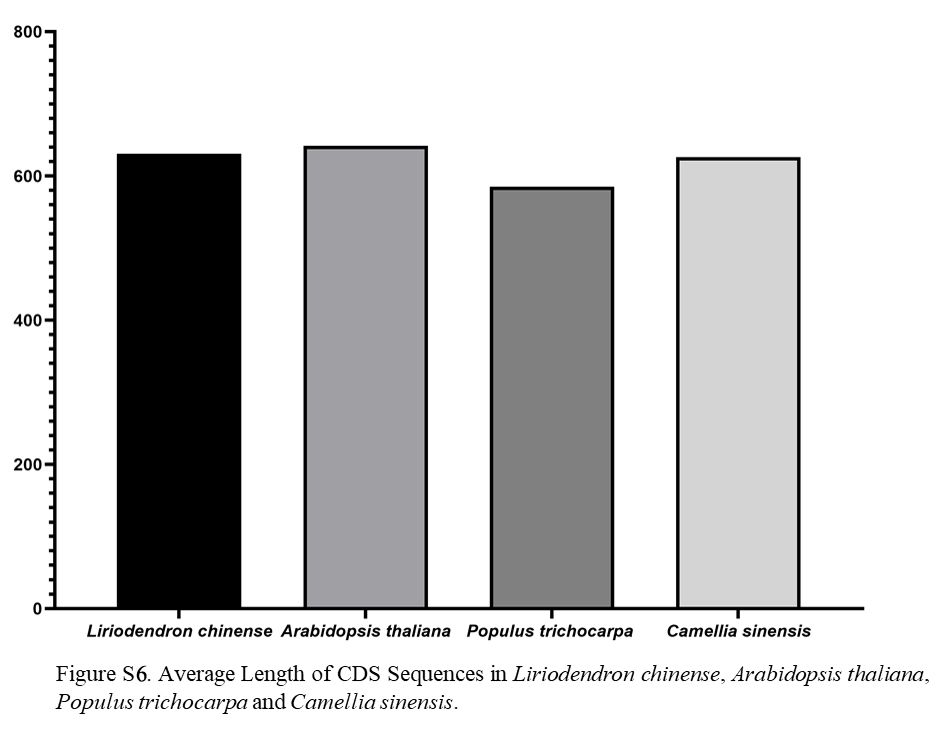

Supplement: Supplementary file 1 [file genes-14-01125-s001.zip › Figure S6.jpg]
